# Parasitoid envenomation alters the *Galleria mellonella* midgut microbiota and immunity, thereby promoting fungal infection

**DOI:** 10.1038/s41598-019-40301-6

**Published:** 2019-03-08

**Authors:** Olga V. Polenogova, Marsel R. Kabilov, Maksim V. Tyurin, Ulyana N. Rotskaya, Anton V. Krivopalov, Vera V. Morozova, Kseniya Mozhaitseva, Nataliya A. Kryukova, Tatyana Alikina, Vadim Yu. Kryukov, Viktor V. Glupov

**Affiliations:** 1 Institute of Systematics and Ecology of Animals, Siberian Branch, Russian Academy of Sciences, Novosibirsk, 630091 Russia; 2 Institute of Chemical Biology and Fundamental Medicine, Siberian Branch, Russian Academy of Sciences, Novosibirsk, 630090 Russia

## Abstract

Gut bacteria influence the development of different pathologies caused by bacteria, fungi and parasitoids in insects. Wax moth larvae became more susceptible to fungal infections after envenomation by the ectoparasitoid *Habrobracon hebetor*. In addition, spontaneous bacterioses occurred more often in envenomated larvae. We analyzed alterations in the midgut microbiota and immunity of the wax moth in response to *H. hebetor* envenomation and topical fungal infection (*Beauveria bassiana*) alone or in combination using 16S rRNA sequencing, an analysis of cultivable bacteria and a qPCR analysis of immunity- and stress-related genes. Envenomation led to a predominance shift from enterococci to enterobacteria, an increase in CFUs and the upregulation of AMPs in wax moth midguts. Furthermore, mycosis nonsignificantly increased the abundance of enterobacteria and the expression of AMPs in the midgut. Combined treatment led to a significant increase in the abundance of *Serratia* and a greater upregulation of gloverin. The oral administration of predominant bacteria (*Enterococcus faecalis, Enterobacter* sp. and *Serratia marcescens*) to wax moth larvae synergistically increased fungal susceptibility. Thus, the activation of midgut immunity might prevent the bacterial decomposition of envenomated larvae, thus permitting the development of fungal infections. Moreover, changes in the midgut bacterial community may promote fungal killing.

## Introduction

Relationships between parasites and hosts can be mediated by endosymbiotic microorganisms from both the parasite and host sides. Especially, endosymbionts may protect hosts against parasitic invaders, whereas parasites are able to manipulate the hosts microbiota directly or through an alteration of host’s immunity^1^. In insects, the gut microbiota plays a substantial role in digestion, regulation of the immune system, detoxification of allelochemicals, protection against pathogens, stress responses, and other vital functions^2–4^. Changes in the structure of the gut microbial community may lead to alterations in the susceptibility to pathogenic microorganisms and to substantial modifications in the development of diseases^5–8^. Some opportunistic pathogens in the gut can demonstrate pathogenic properties under specific conditions, such as the action of abiotic and biotic stress factors, including different infections^5,7–9^. The influence of the gut microbiota on the development of pathogenesis was extensively studied for bacterioses caused by *Bacillus thuringiensis*^5–7,9–12^; however, the impact of the gut microbiota on the progress of these infections is still debated.

The impact of gut bacteria in the development of mycoses is less studied^1^. It is well known that gut bacteria can inhibit fungal growth and sporulation^13,14^. Therefore, germ-free insects can be more susceptible to entomopathogenic ascomycetes after the oral administration of the fungi^15^. Similarly, the oral secretions of bark beetles contain bacterial complexes that inhibit the growth of opportunistic fungi in galleries^16^. Kabaluk and coauthors^17^ showed that bacteria from the genera *Pantoea, Pandoraea*, *Nocardia* and *Mycobacterium* impeded the development of infection by the fungus *Metarhizium brunneum* in field populations of wireworms. Aphid endosymbionts *Spiroplasma* and *Ricketsia* may protect against entomophtoralean fungus *Pandora neoahidis*^18^. On the other hand, some authors demonstrated synergy between gut bacteria and fungi. In particular, Wei and coworkers^8^ showed that topical infection with *Beauveria bassiana* reduced the taxonomical diversity of the mosquito gut microbiota by decreasing its due to the mass proliferation of *Serratia marcescens*, which in turn increases susceptibility to the fungus. An additive effect between species of the gut bacteria *Pseudomonas* and entomopathogenic ascomycetes was demonstrated in locusts^19^. Moreover, in many cases, fungal infections lead to the bacterial decomposition of the killed insects. These phenomena are usually stipulated by exposure of insects to high conidia doses or changes in production of fungal toxins^1,20^.

Studies on the relationship between parasitoids and the host insect microbiota have been performed mainly in host-parasite systems involving endoparasitoids and aphids. In most cases, these investigations concentrate on the beneficial effects of endosymbionts^21^. It was shown that an aphid facultative symbiont, namely, the enterobacteria *Hamiltonella defensa*, protects aphids against the endoparasitoid *Aphidius ervi*, leading to the death of the endoparasitoid larvae inside the aphid body^22–24^. Similarly, the enterobacteria *Regiella insecticola* provides aphids with protection against the endoparasitic wasps *Aphidius colemani* and *A. ervi*^25,26^. In contrast, a positive correlation between the frequency of infection by bacterial symbionts and parasite pressure was shown for a system including the enterobacteria *Arsenophonus*, the endoparasitoid *Psyllaephagus bliteus* and the host *Glycaspis brimblecombei*^27^. Relationships between host symbiotic bacteria and ectoparasitoids, as well as the influence of the parasitoids on the structure of the host microbiota have not been studied.

It is important to notice that the way how parasitic organisms affect host’s immunity and gut microbiota was mainly studied on models including one host and one parasite. Influence of co-infections on the physiological parameters was less studied. However, in real conditions insects are exposed to a complex of parasites, commensals, mutualists that lead to complicated interrelation between them and are mediated by host immune reaction^8,28,29^. Triple host-parasite system, consisting of parasitoid *Habrobracon hebetor* (Hymenoptera: Braconidae), entomopathogenic ascomycete (e.g., *Beauveria*) and wax moth *Galleria mellonella* exhibits one of the intriguing system for parasitological study.

*Habrobracon hebetor* is a gregarious ectoparasitoid having a wide host range which includes lepidopterans from different families^30^. The parasitoid females paralyze host larvae through envenomation. The paralysis of hosts is caused by the disruption of glutamatergic receptors on presynaptic membranes^31^. In addition, envenomation leads to a dramatic decrease in both cellular and humoral immunity in hosts, specifically in the wax moth^32,33^. Importantly, *H. hebetor* females paralyze many host individuals, and parasitoid larvae do not completely consume their hosts. Envenomated insects do not survive; they are eventually colonized with different microorganisms, primarily bacteria. However, if envenomated larvae were topically inoculated with the entomopathogenic ascomycetes *Beauveria, Isaria*, or *Metarhizium* (even at extremely low doses) they were successfully colonized by the fungi^34,35^. Interestingly, envenomated wax moth larvae were thousands of folds more susceptible to fungal infection compared to nonenvenomated larvae^34^, and the susceptibility was increased to pathogenic but not opportunistic fungi^36^. Importantly, arrest of gut peristalsis occurred under the influence of the venom. We hypothesized that envenomation and the absence of gut peristalsis led to the disruption of the microbiota, changes in the gut immune responses, and the proliferation of some endosymbiont bacteria, while topical fungal infection can prevent the development of bacteria. Additionally, we suggested that changes in the predominant bacterial taxonomic groups could be one of the factors leading to the increase in the susceptibility of wax moth to entomopathogenic fungi.

It is known that changes in the bacterial community are correlated with the immune response in the midgut, e.g.^8,11,37,38^. The humoral defenses of the wax moth include at least 18 antimicrobial peptides, especially gloverin, gallerimycin and galiomycin, which exhibit antibacterial and/or antifungal activity^39,40^. The inducible metalloproteinase inhibitor (IMPI) inhibits the activity of metalloproteinases secreted by many pathogenic organisms as virulence factors, thus controlling the degradation of immune-relevant polypeptides in the infected host^41,42^. The heat shock protein 90 (Hsp90), caspase protein (CASP), glutathione peroxidase (GSH PX) and macrophage migration inhibitory factor (MIF) genes of *Galleria mellonella* contain conservative domains that are evolutionarily ancient and are found in both vertebrates and invertebrates^43–48^. These proteins play regulatory and chaperone roles in innate immune, metamorphosis-related and ROS management processes^45–48^. The expression of these genes may be induced during cell membrane rupture in response to destructive processes, such as the proteinase-mediated lysis caused by fungal and bacterial infections in insects^45,46,49^. Thus, assays of the expression of these genes could highlight the processes occurring in the wax moth midgut in response to envenomation and fungal infection.

In the present work, we studied changes in the microbiota, humoral immunity and stress response of the wax moth midgut in response to *H. hebetor* envenomation and *B. bassiana*-induced mycosis alone and in combination. In addition, we estimated the impact of the predominant cultivable bacteria in the efficacy of *B. bassiana* infection.

## Results

### Bioassay

**Figure 1:**
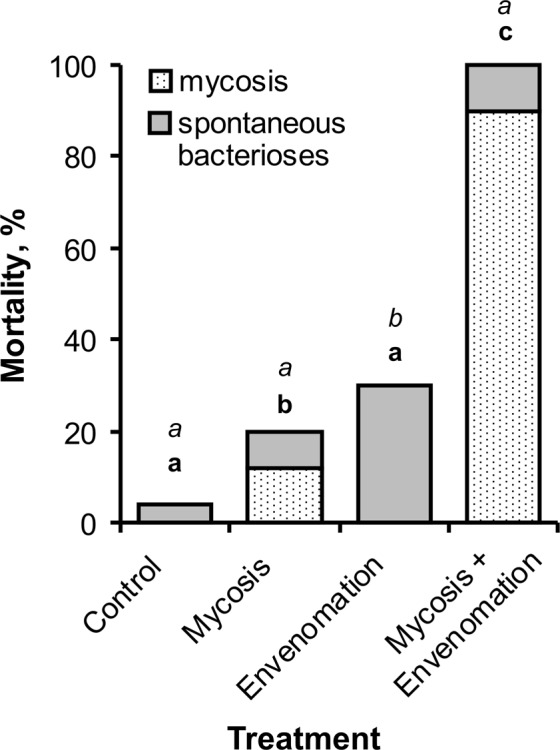
Mortality rate of wax moth larvae for 12 days post envenomation by *Habrobracon hebetor* or infection with *Beauveria bassiana* (10^6^ conidia/ml) alone or in combination. Fifty larvae were used for each treatment. Identical italicized letters indicate nonsignificant differences in the bacteriosis incidence between treatments, and identical bold letters indicate nonsignificant differences in death due to *B. bassiana* infection between treatments (χ^2^ > 2.3; P > 0.08).

The topical infection of nonenvenomated larvae with *B. bassiana* led to 12% mortality due to the mycosis over 12 days (Fig. 1), which significantly differed from that of intact (control) larvae (χ^2^ = 6.4, P = 0.01). Treatment of envenomated larvae with *B. bassiana* led to 90% mortality due to the fungus (χ^2^ = 60.1, P < 0.0001, compared to infected nonenvenomated larvae). The development of spontaneous bacterioses was higher in envenomated but noninfected larvae (30%) than in larvae subjected to other treatments (χ^2^ > 6.3, P < 0.01). Fungal infection caused a significant decrease in the incidence of bacteriosis among envenomated larvae, to 10% (χ^2^ = 6.25, P = 0.01).

### Changes in midgut bacterial communities

Classification based on 16S rRNA gene sequencing revealed 176 operational taxonomical units OTUs grouped in 117 taxa at the genus level, 72 taxa at the family level and 11 taxa at the phylum level (see Appendix A). Over all treatment groups, the main phyla were Proteobacteria (62.9%), with a predominance of Enterobacteriaceae, and Firmicutes (35.7%), with a prevalence of Enterococcaceae. The envenomation of the wax moth by the parasitoid dramatically decreased the diversity of the bacterial community (Table 1). In particular, a large decrease in the number of OTUs, genera and families (H_1,15_ > 11.3, P < 0.001), along with a decreasing tendency in the Shannon index (H_1,15_ = 2.8, P = 0.09) were noted in response to the venom. The effects of the fungus on the taxonomic diversity were not significant (H_1,15_ < 0.3, P > 0.6). However, an increasing tendency in the Shannon index was observed in response to mycosis (H_1,15_ = 3.2, P = 0.07) due to the leveling of the abundances between the predominant groups of bacteria (see below).

**Table 1:** Diversity characteristics of the microbiota in wax moth midguts at 72 hours post envenomation by *Habrobracon hebetor* or infection with *Beauveria bassiana* alone or in combination.

	OTUs	Genera	Families	Shannon Index*
Control	54 ± 2.9^a^	43 ± 2.4^a^	31 ± 1.4^a^	0.9 ± 0.32^ab^
Mycosis	45 ± 4.6^ac^	37 ± 3.9^ac^	28 ± 2.9^a^	1.4 ± 0.44^a^
Envenomation	13 ± 4.6^b^	11 ± 3.7^b^	10 ± 2.8^b^	0.3 ± 0.11^b^
Mycosis + Envenomation	15 ± 2.3^bc^	14 ± 1.9^bc^	11 ± 1.0^b^	0.9 ± 0.21^ab^

*The Shannon index was calculated for the genus level.

The standard error (±SE) was calculated for four replicates. Identical letters indicate nonsignificant differences among treatments (Dunn’s post hoc test: P > 0.05).

**Figure 2:**
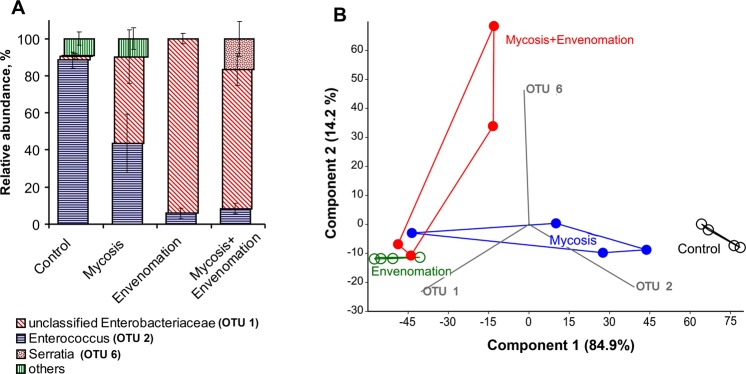
Alteration of the midgut microbiota composition in wax moth larvae at 72 hours post envenomation by *Habrobracon hebetor* or infection with *Beauveria bassiana* alone or in combination. (**A**) Histogram showing changes at the genus level. Vertical bars show the standard error (±SE) calculated from four biological replicates. (**B**) Principal component analysis of microbial communities for the OTU level.

Three OTUs dominated in the investigated samples: OTU 1 – unclassified Enterobacteriaceae, OTU 2 − *Enterococcus* and OTU 6 – *Serratia* (Fig. 2A). Control (untreated) larvae were characterized by the prevalence of *Enterococcus* (88 ± 4.7%) and the low relative abundance of enterobacteria (<8.2%) in the midgut (Fig. 2A). Envenomation led to significant alterations in the microbiome structure caused by an increase in the proportion of Enterobacteriaceae and a decrease in the proportion of *Enterococcus* (H_1,15_ = 7.45, P = 0.006). A trend of enhancement in the abundance of enterobacteria was noted after treatment with the fungus alone, but the difference were not significant relative to that of the control group (Dunn’s test, P = 0.075). However, an increase in the *Serratia* abundance was observed during acute mycosis development in envenomated larvae (Dunn’s test, P < 0.04, compared to other treatments). The principal component analysis of the microbial communities showed that the first component explained 84.9% of the variation in the data, which was due to changes in the relationship between enterobacteria (OTU 1) and enterococci (OTU 2) (Fig. 2B). Clear clustering is observed between the envenomated and nonenvenomated (control) larvae. Samples from infected larvae occupy intermediate locations in the plot. The second principal component explained only 14.2% the variation, which was caused by an increase in Otu 6 (*Serratia*) in envenomated and infected larvae. Notably, a large reduction in the relative abundance of the other taxa was observed in envenomated larvae (Fig. 2A, Appendix A). We hypothesized that this phenomenon was caused by the mass proliferation of enterobacteria; therefore, we estimated the CFUs of the predominant midgut bacteria using selective media.

### Abundance, identification and antagonistic properties of cultivable bacteria

**Figure 3:**
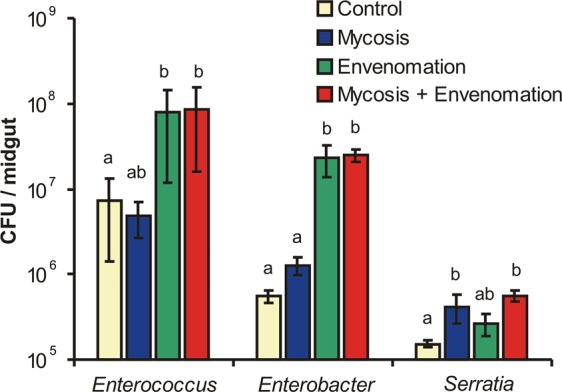
Changes in the CFU counts of cultivable bacteria from the midgut of wax moth larvae at 72 hours post envenomation by *Habrobracon hebetor* or infection with *Beauveria bassiana* alone or in combination. Selective media for *Enterococcus* (modified bile esculin azide agar), *Enterobacteriaceae* (Endo agar) and *Serratia* (Serratia differential medium) were used. Vertical bars show the standard error (±SE) calculated from seven replicates (*Enterococcus*), nine replicates (*Enterobacter*) or three replicates (*Serratia*). Identical letters indicate nonsignificant differences among treatments (Dunn’s post hoc test: P > 0.05).

The plating of midguts on selective media showed an increase in the CFUs of all dominant bacteria (*Enterococcus, Enterobacter* and *Serratia*) in response to envenomation (Fig. 3). Importantly, the CFU count of *Enterococcus* was enhanced 10- to 12-fold, but the CFU count of *Enterobacter* was enhanced 42- to 45-fold in envenomated (or both infected and envenomated) insects compared to the CFU counts of those bacteria in larvae that were untreated or only infected with the fungus (envenomation effects: H_1,27_ = 8.1, P = 0.004 for *Enterococcus* and H_1,35_ = 19.9, P < 0.0001 for *Enterobacter*). Fungal infection had no significant effects on the CFUs of *Enterococcus* and *Enterobacter* (H < 1.6, P > 0.26); however, it did impact the *Serratia* abundance. Specifically, the development of mycosis led to an increase in the *Serratia* CFU count in envenomated and nonenvenomated larvae (H_1,11_ = 5.8, P = 0.016).

Sequencing of the 16S rRNA region showed that the sequences of seven isolates of *Enterococcus* were identical to each other and were characterized by 100% sequence identity to the majority of *E. faecalis* strains, especially the type strain of JSM5803^T^ (Appendix B, Fig. S1). Furthermore, five isolates of *Enterobacter* also had a molecular haplotype (Appendix B, Fig. S2) identical to that of strain M. pstv. 10.4 (KM108494) *E*. *hormaechei* (*E*. *cloacae* complex), which was isolated from water containing *Aedes aegypti* larvae^50^. The sequences of the investigated isolates differed by one single nucleotide position (SNP) from that of *E*. *hormaechei* 69–907 R (KX828280), which was isolated from the gut of the fish *Oreochromis niloticus*, and by one SNP from that of *E*. *xiangfangensis* strain 10–17 ^T^ (HF679035), which was isolated from Chinese traditional sourdough^51^. Meanwhile, the sequence of the type strain of *E*. *hormaechei* CIP 103441 (AJ508302) differed from those of our isolates by four SNPs. Thus, due to the ambiguous phylogenetic placement of our *Enterobacter* isolates and the sequence similarity to both species, we identified the isolates as *Enterobacter* sp. The sequence was deposited in GenBank under the accession number MH685399. The sequences of two *Serratia* isolates were identical to each other (Appendix B, Fig. S2) and differed by one SNP from that of the type strain *S*. *marcescens* subsp. *marcescens* DSM 30121 ^T^ (AJ233431), which was isolated from pond water^52^ and from the type strain of *S. marcescens* subsp. *sakuensis* KRED^T^ (AB061685), which was isolated from waste water^53^. The sequence was deposited in GenBank under the accession number MH685393. The *E. faecalis* and *S. marcescens* sequences were 100% identical to those of OTU 2 (*Enterococcus*) and OTU 6 (*Serratia*), respectively. The sequences of *Enterobacter* sp. demonstrated 99.4% identity with that of OTU 1 (unclassified *Enterobacteriaceae*).

The analysis of antagonistic interactions between the investigated bacteria and *B. bassiana* showed that the fungus did not inhibit the growth of the bacteria. Similarly, *Enterobacter* sp. had no effect on fungal growth. *E. faecalis* and *S. marcescens* inhibited *B. bassiana* mycelial growth by 4 ± 0.4 mm and 8 ± 2.0 mm, respectively (Appendix B, Fig. S3).

### Gene expression after envenomation and fungal infection

**Figure 4:**
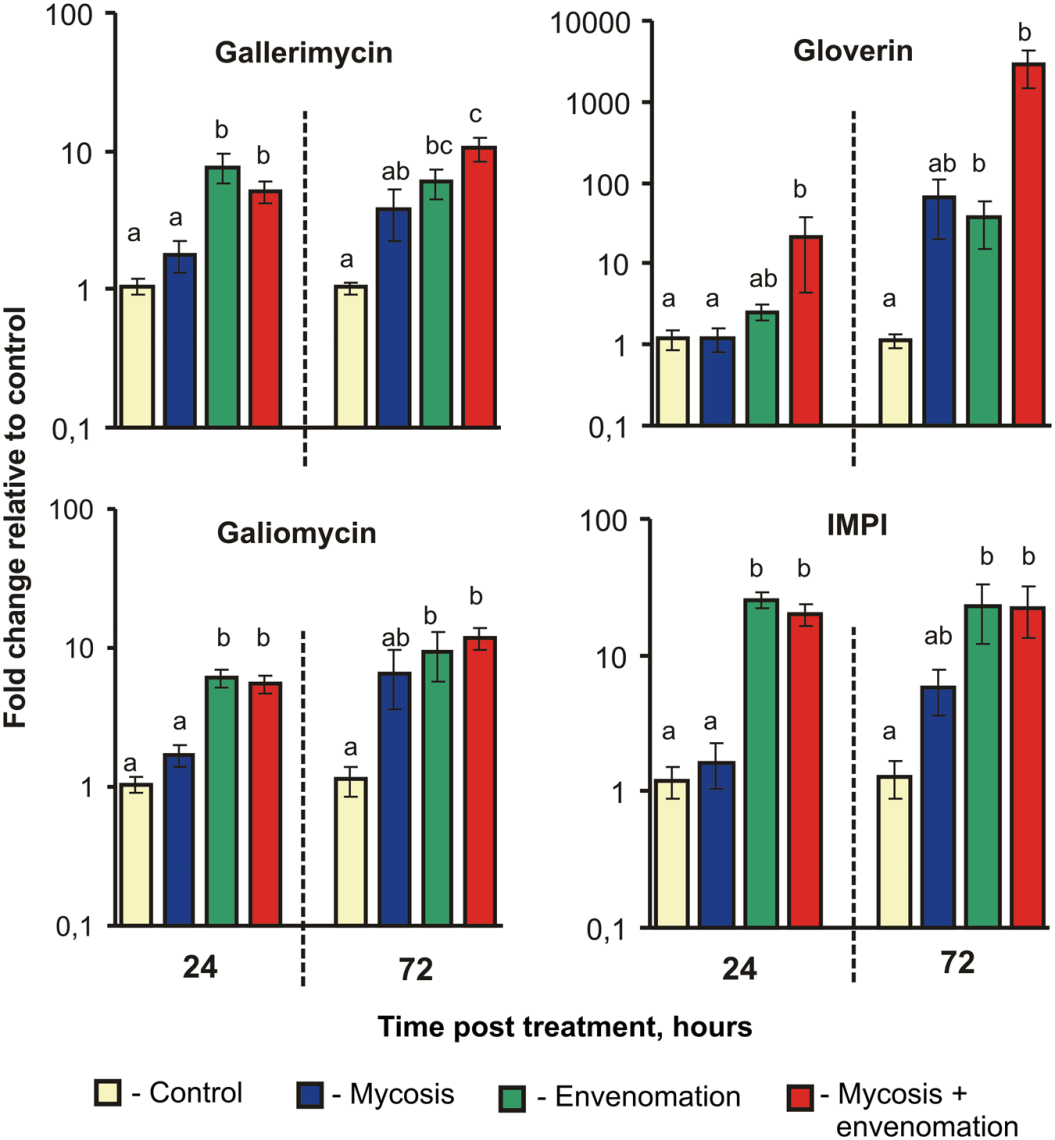
Relative expression of antimicrobial peptide genes (gallerimycin, gloverin, and galiomycin) and IMPI in response to *Habrobracon hebetor* envenomation and *Beauveria bassiana* infection, normalized to the expression of reference genes, at two time points – 24 and 72 hours. The results are expressed as the mean fold change in relative expression compared to that of the control at each time point. The bars show the standard error (±SE) calculated from six biological replicates. Identical letters indicate nonsignificant differences among treatments at each time point (Dunn’s post hoc test: P > 0.05).

There were similar trends in the expression of AMPs and IMPI in the midguts in response to parasitoid envenomation and mycosis development (Fig. 4). On the first day post treatment, an upregulation in the expression of all investigated AMPs as well as the IMPI gene was observed in response to envenomation (H_1,23_ = 15.0, P = 0.0001 for galiomycin; H_1,23_ = 17.2, P < 0.0001 for gallerimycin; H_1,23_ = 8.3, P = 0.003 for gloverin; and H_1,23_ = 17.3, P < 0.0001 for IMPI gene expression). Fungal infection did not lead to significant changes in the expression of these genes on the first day (H_1,23_ < 0.26, P > 0.60). On the third day post treatment, we observed an increase in the expression of all AMPs and IMPI (H_1,23_ > 6.8, P < 0.01) in response to envenomation. Mycosis caused a significant increase in gloverin gene expression (H_1,23_ = 4.6, P = 0.03), along with similar effects on galiomycin and gallerimycin expression, at this time point, but the differences in the expression of these two genes were not significant (H_1,23_ = 3.0, P = 0.08 and H_1,23_ = 2.8, P = 0.09, respectively). A greater degree of upregulation was noted for gloverin than for the other genes in response to the combination of fungus and venom (2,900-fold compared to the control, Dunn’s test, P < 0.001).

**Figure 5:**
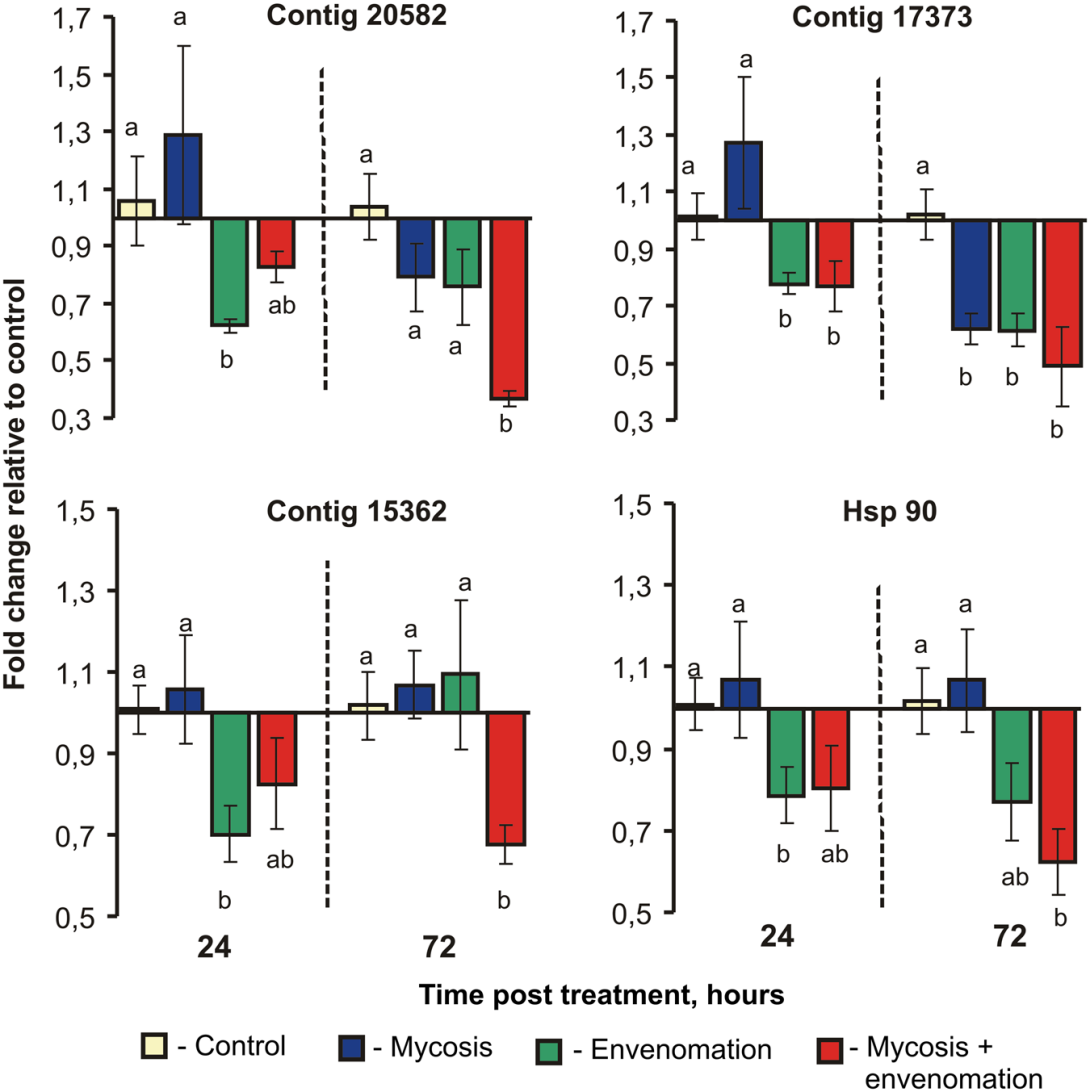
Relative expression of ROS-, inflammation- and stress-related genes (Contig 15362 – CASP, Contig 20582 – MIF, Contig 17373 – GSH PX and HSP 90) in response to *Habrobracon hebetor* envenomation and *Beauveria bassiana* infection, normalized to the expression of reference genes, at two time points – 24 and 72 hours. The results are expressed as the mean fold change in relative expression compared to that of the control at each time point. The bars show the standard error (±SE) calculated from six biological replicates. Identical letters indicate nonsignificant differences among treatments at each time point (Dunn’s post hoc test: P > 0.05).

We observed small (no greater than 3-fold) changes in the expression of ROS-, inflammation- and stress-related genes in wax moth midguts; however, the effects of envenomation were significant (Fig. 5). At both time points, envenomation led to the downregulation of Contig 15362 – CASP (H_1,23_ > 3.9, P < 0.04), Contig 20582 – MIF (H_1,23_ > 6.5, P < 0.01), Contig 17373 – GSH PX (H_1,23_ > 3.5, P < 0.06) and Hsp 90 (H_1,23_ > 4.56, P < 0.03). Fungal infection led to the downregulation of MIF (H_1,23_ = 6.8, P = 0.009) and GSH PX (H_1,23_ = 4.2, P = 0.04) in envenomated and nonenvenomated larvae on the third day post treatment. A slight trend towards a decreased expression of the genes was observed on the third day of the experiment in response to the combined treatment, compared to envenomation or mycosis alone. However, significant factor interactions were not observed (H_1,23_ < 2.0, *P* > 0.16 for MIF, GSH PX and Hsp 90, and H_1,23_ < 3.5, *P* > 0.06 for CASP).

### Bioassay of the cultivable bacteria and the fungus

**Figure 6:**
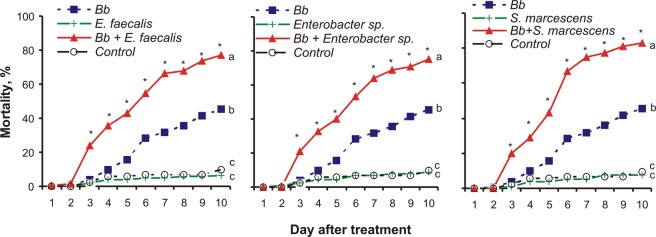
Dynamics of wax moth larvae mortality after the oral administration of midgut bacteria (10^8^ cells/3 g of food) and topical infection with the fungus *Beauveria bassiana* (*Bb*) (4 × 10^6^ conidia/ml). Data are pooled from three experiments. Total n = 73 larvae for control, 177 larvae for *Bb*, 140 larvae for *E. faecalis*, 137 larvae for *Enterobacter* sp., 135 larvae for *S. marcescens*, 155 larvae for *Bb* + *E. faecalis*, 172 larvae for *Bb* + *Enterobacter* sp. and 178 larvae for *Bb* + *S. marcescens*. Different letters show significant differences in mortality dynamics between treatments (log-rank test, χ^2^ > 55.1, P < 0.0001). *synergy between fungi and bacteria, calculated as described by Robertson & Preisler^78^ (Appendix B, Table S1).

The oral administration of cultivable midgut bacteria (*E. faecalis, Enterobacter sp*. and *S. marcescens*) did not result in wax moth larvae mortality (log-rank test: χ^2^ < 0.7, P > 0.38, compared to the uninfected control). However, synergy between these bacteria and the fungus was observed after combination treatment (Fig. 6). The synergy was observed from 3 to 10 days after infection (χ^2^ > 25.3, P < 0.001). Though the effects in response to combined treatment with *B. bassiana* and tested bacteria were similar (a 30–38% increase in the total mortality and a 2-fold decrease in the survival time (ST_75_) after mixed infection compared to the mortality dynamics of fungal infection alone), the greatest synergy was observed between *B. bassiana* and *S. marcescens* (Appendix B, Table S1). All larvae that were treated with the combination of fungus and bacteria, were overgrown with *B. bassiana* if they died on or after day 3 post treatment.

## Discussion

The performed study showed that the envenomation of wax moth larvae by the ectoparasitoid *H. hebetor* leads to the uncontrolled proliferation of midgut bacteria and to a significant predominance shift from gram-positive enterococci to gram-negative enterobacteria. However, activation of midgut immunity (AMPs and IMPI) occurred in envenomated insects. We propose that the activation of midgut immunity can prevent the bacterial decomposition of envenomated larvae for several days, thus permitting the development of fungi and/or parasitoid larvae. Moreover, changes in the midgut bacterial community lead to an increase in the susceptibility to the fungus *B. bassiana* after topical infection.

The lepidopteran larvae gut is characterized by the high pH, simple structure, and rapid transit time^54^. The continuous flow of the gut contents causes the regular renewal of the microbiome content and the cells of the peritrophic membrane. Thus, the paralysis of gut peristalsis should disrupt the bacterial balance, pH and absorption functions of the gut cells. Under normal conditions, the gut microbiome of *G. mellonella* laboratory lines consists mainly of gram-positive bacteria of the genus *Enterococcus*^11,37,38^, which agrees with the results of our study. The inhibition of peristalsis can induce competition among bacteria. The analysis of 16S rRNA showed a decrease in the taxonomical diversity and the replacement of *Enterococcus* by gram-negative *Enterobacteriaceae* in the midgut in response to parasitoid envenomation. Interestingly, this shift in the community structure from gram-positive to gram-negative bacteria has been observed during other toxicoses. In particular, the replacement of *Enterococcus* by *Enterobacter* in wax moth midguts during *B. thuringiensis* infection was demonstrated by Dubovskiy and coauthors^11^. The depletion of community diversity and the increase in the abundance of gram-negative bacteria (*Stella* sp.) in *Lymantria dispar* after *B. thuringiensis* infection was also reported by investigators^55^. Similar results were obtained in response to the topical infection of adult *Anopheles stephensi* mosquitoes with *B. bassiana*. In this case, mycosis led to a significant decrease in the bacterial diversity and a shift of the dominant bacteria from *Firmicutes* to *Proteobacteria*^8^. In particular, acute mycosis led to an increase in *S. marcescens* abundance, which promotes fungal infection in mosquitoes^8^. In the present work we also found an increase in *Serratia* abundance during the development of acute fungal infection in envenomated larvae.

In general, changes in the CFU count of the dominant bacteria are correlated with the 16S RNA data: the CFU count of *Enterobacter* sp. was increased more than that of *E. faecalis* in response to envenomation. Moreover, fungal infection led to an increase in *S. marcescens* CFUs. On the basis of these data, we suggest that of the decrease in the diversity of the wax moth microbiome in response to envenomation occurs due to the mass proliferation of enterobacteria. Interestingly, we did not find *Enterococcus mundtii*, though several researchers noted that *E. mundtii* is one of the predominant bacteria in the *G. mellonella* midgut^37,38^. Most likely, the presence of the bacteria is dependent on the origin of the wax moth strain or on the diet.

It is known that an imbalance in the gut microbial components triggers local AMP expression, which provides an immune response against opportunistic infections and controls the balance of gut symbionts^56,57^. *Enterobacter* are classified as facultative anaerobes that can act as opportunistic pathogens in immunosuppressed hosts^54,58^. Therefore, any disruptions in this balanced system activated defense systems and the synthesis of AMPs. Importantly, envenomated insects respond by expressing AMPs and IMPI upon changes in the bacterial community. The highest activation has been observed for gloverin, which possess antibacterial activity exclusively against gram-negative bacteria^59^. IMPI is able to inactivate bacterial metalloproteases^60^, and an increase in its expression after envenomation was also confirmed in studies on bacterioses^11,61^. In addition, we found an enhanced expression of gallerimycin and galiomycin after envenomation. These proteins are mainly antifungal components^40^, but an increase in the expression of their genes in the wax moth was revealed in response to various bacterial treatments^11,61,62^. Furthermore, the level of expression of these genes in the *G. mellonella* midgut was positively correlated with the diversity of the diet and the CFU count of intestinal bacteria^38^. Moreover, it was shown that the antifungal component is able to act synergistically with other AMPs against bacteria^63^. Previously, Wei and coworkers^8^ suggested that alterations in the bacterial community in response to mycosis development are caused by the downregulation of the expression of AMPs and dual oxidase in the mosquito midgut. We did not observe the downregulation of AMPs in response to fungal infection. In contrast, a significant increase in gloverin gene expression and increasing trends in galiomycin and gallerimycin expression were found. Thus, the change in AMP expression in our system is the effect (symptom) but not the cause of dysbiosis.

We revealed weak changes and downregulation in the expression of ROS-, inflammation- and stress-related genes compared to the changes in AMP expression in response to envenomation and mycosis. This result may indicate the absence of destructive processes caused by oxidative/inflammatory damage during the investigated period (1–3 days post envenomation and infection). It is known that macrophage migration inhibitory factor (Contig 20582) and glutathione peroxidase (Contig 17373) are members of the antioxidant system^43,44^. The expression of the genes encoding Hsp 90 and caspase-like protein (Contig 15362) were indicative of destructive processes in the gut cells^64^. The active synthesis of Hsp 90 often occurs in tandem with caspase-like protein and indicates cell disruption by apoptotic or necrotic pathways in insects^64^. The downregulation of genes after envenomation alone or after envenomation and fungal infection together can be caused by general attenuation and starvation in insects.

We suggest that the activation of AMPs and the absence of midgut damage can prevent septicemia and bacterial decomposition of larvae for at least several days after envenomation. This time period could allow parasitoid larvae to finish feeding, since the developmental time from egg to pupa is only 5–7 days. Furthermore, the successful development of *B. bassiana* in envenomated larvae requires 3–7 days, even after infection with low doses^34^. Midgut bacteria can inhibit *B*. *bassiana* growth, but fungal colonization forestalls the development of systemic bacteriosis. It seems that the gut immune reaction of the host is beneficial for both parasitoids and fungi in the investigated system.

Interestingly, the oral administration of three cultivable bacteria, namely, *E. faecalis, Enterobacter* sp. and *S. marcescens*, did not cause larval mortality but significantly increased larval susceptibility to *B. bassiana*. Thus, despite the antagonistic properties of the bacteria towards *B. bassiana*, they are able to increase larval mortality after topical fungal infection. The obtained data confirm that the gut microbiota can strongly promote fungal killing activity^8^. The mechanism of the observed synergy is unclear. Previously, we showed that one of the main antifungal reactions – encapsulation – was decreased in wax moth hemolymph after envenomation by *H. hebetor*^32,35^. The decrease in this parameter can be caused by either the direct action of the venom on hemocytes^33^ or through the influence of midgut bacteria. Krams and coworkers^38^ revealed that the decrease in the encapsulation rate in wax moth larvae correlates with the increase in gut bacteria abundance. These researchers^38^ hypothesized that there is antagonistic cross-regulation between different components of the immune system – AMP expression in the midgut and encapsulation in the hemolymph. Possibly, the disturbance of the symbiotic balance under a high load of bacteria leads either to changes in the prioritization of immune responses between the midgut, integument and hemocoel, or to the suppression of antifungal immune reactions by bacterial metabolites, as shown by Park & Kim^28^ in the *Spodoptera exigua* – *Xenorhabdus nematophila – B. bassiana* system. Reasons for the synergy between the midgut bacteria and *B. bassiana* should be revealed in future studies.

Thus, the work has shown a substantial impact of the host gut microbiota and gut immunity on interaction in three-component system host - parasitoid - entomopathogenic fungus. Though both parasites do not affect the gut directly, they cause significant alterations in gut physiology. Parasitoid envenomation significantly decreases the midgut bacterial diversity due to the mass proliferation of enterobacteria. In addition, fungal infection increases abundance of opportunistic pathogen *Serratia*, particularly in envenomated insects. However, envenomated and infected insects respond by enhancing AMP expression, which can confine bacteria within the gut lumen and permit the development of fungus. Moreover, the additional load of cultivable bacteria in the gut promotes the development of fungal infection. Previously, many researchers considered mechanisms of synergy between entomopathogenic fungi and various intestinal or systemic toxicants (including parasitoid venoms) based on different changes in humoral and cellular immune reactions^35,65–70^. However, the impact of the toxicants on microbial communities was insufficiently understood. Our study shows that changes in the gut microbiota can be an important reason for the synergy. Further investigations could focus on the crosstalk between gut bacteria and fungi penetrating through the integument as well as on the interaction between fungi, toxicants and gut bacteria. Understanding these interactions may lead to new strategies for the biological control of insect pests.

## Material and Methods

### Insects and fungal material

Insects and fungi from the Siberian Zoological Museum of the Institute of Systematics and Ecology of Animals SB RAS (Novosibirsk) were used for the experiments. A laboratory population of *G. mellonella* (Siberian line) was maintained at 28 °C and 60% RH on artificial medium containing 22.5% corn meal, 12.5% beeswax, 12.5% honey, 12.5% glycerol, 12.5% milk solids, 10% wheat flour, 5% yeast and 12.5% water. *H. hebetor* was grown on the later instars of *G. mellonella* larvae^32^. Adult parasitoids were maintained at 28 °C with a photoperiod of 14:10 h (light:dark) on a diet of a 12% water-honey solution. The Sar-31 *Beauveria bassiana* s.s. isolate was used in the study. The conidia for infection were grown on Sabouraud dextrose agar (SDA) at 26 °C for 10 days, harvested by scraping, air-dried at 25 °C for 1 week and stored at 4 °C. For infections, conidia were suspended in sterile 0.003% Tween 20 and vortexed for 1 min. Concentrations of conidia were determined using a Neubauer hemocytometer.

### Envenomation and inoculation procedures

Two *H. hebetor* females were placed for 10 h in 90 mm Petri dishes with ten fifth-instar larvae of *G. mellonella* for envenomation. Then, eggs of the parasitoid were washed off of paralyzed larvae with distilled water. Envenomated and nonenvenomated larvae were dipped in either a water-Tween suspension of *B. bassiana* (1.0 × 10^6^ conidia/mL), or in a conidia-free water-Tween solution for 10 s. Then, the larvae were placed in 90 mm Petri dishes (10 larvae per dish) with 80 mm discs of filter paper moistened by sterile water (1 mL per disk). Petri dishes with insects were maintained in a climatic chamber (KBWF 240, Binder, Germany) at 28 °C and 75% RH. Larval mortality was estimated over 12 days. Cadavers were placed in moisture chambers to confirm mortality due to *B. bassiana*.

### DNA extraction and Illumina sequencing of wax moth midguts

Seventy-two hours after fungal inoculation and/or envenomation, wax moth larvae were surface-sterilized with 70% ethanol and were dissected. Midguts with content were extracted and frozen in liquid nitrogen (5 midguts = one sample). Four samples of each treatment were used for analysis. DNA was isolated by a PowerSoil DNA Isolation Kit (Mo Bio). The V3-V4 region of the 16S rRNA genes was amplified with the primer pair 343 F (5′-CTCCTACGGRRSGCAGCAG-3′) and 806 R (5′-GGACTACNVGGGTWTCTAAT-3′) combined with Illumina adapter sequences^71^. The PCR conditions were as described previously^72^. A total of 200 ng of PCR product from each sample was pooled and purified with a MinElute Gel Extraction Kit (Qiagen). The 16S libraries were sequenced with 2 × 300 bp paired-end reagents on a MiSeq (Illumina) platform in the SB RAS Genomics Core Facility (ICBFM SB RAS, Novosibirsk, Russia). Raw sequences were analyzed with the UPARSE pipeline^73^ using USEARCH v8.1.1861. The UPARSE pipeline included paired read merging, read quality filtering, length trimming, identical read merging (dereplication), singleton read discarding, chimera removal and OTU clustering using the UPARSE-OTU algorithm. The OTU sequences were assigned a taxonomy using SINTAX^74^. Additionally, OTUs classified as chloroplasts were removed. The final data set contained 176 OTUs (308819 reads). All the rarefaction curves had a tendency to approach the saturation plateau, which indicated a reasonable volume of the sequenced reads (Appendix B, Fig. S4). Raw MiSeq reads were deposited in GenBank under the project accession number PRJNA479958 and the sequence read archive (SRA) accession number SRP152548.

### Abundance and molecular identification of cultivable bacteria

Seventy-two hours post treatment, midguts were isolated as described in the previous section and homogenized in 1 mL of 0.1 M phosphate buffer (one sample = 3 midguts). Then, the suspension was diluted to 10^−2^, 10^−3^, 10^−4^ and 10^−5^. One-hundred-microliter aliquots of the 10^−3^ to 10^−6^ midgut dilutions were inoculated onto the surface of media selective for *Enterococcus*, *Enterobacteriaceae* and *Serratia*, namely, modified bile esculin azide agar (HiMedia, India), Endo agar (HiMedia, India), and Serratia differential medium (HiMedia, India), respectively. Petri dishes were incubated at 28 °C for 72 h. Then, enumeration of the colonies on each medium was performed. Representative colonies were selected and passaged three times under the same conditions to select pure strains. Colonies were suspended in sterile water and heated to 95 °С for 5 min, and cellular debris was precipitated by centrifugation. Then, supernatants were used as templates for further PCR and sequencing. Bacterial strains were identified by sequencing a 1308 bp fragment of the 16S rRNA gene using primers 8 F 5′-AGRGTTTGATCCTGGCTCA-3′ and 1350 R 5′-GACGGGCGGTGTGTACAAG-3′, as described previously^75^. The sequences of the 16S rRNA genes of the selected strains were deposited in the GenBank database under accession numbers MH685393 and MH685399. The newly obtained and GenBank-available reference sequences (http://www.ncbi.nlm.nih.gov) were aligned in BioEdit, resulting in an alignment of the genes. To perform phylogenetic analysis, MEGA 7.0 software^76^ was used.

### Antagonism between the fungus and the cultivable bacteria

The antagonistic interaction between *B. bassiana* and cultivable gut bacteria was studied on artificial media by the dual culture method. Plugs (8 mm in diameter) of tryptose agar (Ferak, Germany) with 2-day-old bacterial cultures were placed on fungal lawns freshly plated on SDA medium in 90 mm Petri dishes. The effect of fungi on bacterial growth was assessed in a similar manner: plugs of SDA medium with 4-day-old fungal cultures were placed on tryptose agar medium with freshly plated bacteria. The inhibition zone formed between the fungus and the bacteria was measured on day 4 of dual cultivation.

### Gene expression in the midgut

Wax moth midguts were extracted at 24 and 72 h post treatment, dissected in PBS and cleaned and washed to remove the contents of the gut lumen. After cleaning, midguts were immediately frozen in liquid nitrogen and stored at −80 °C. Before extraction, samples were lyophilized at −65 °C and 500 mTorr for 12 h and homogenized with micropestles in liquid nitrogen. Ten midguts (larvae from one Petri dish) were pooled in one sample, and this sample was used as a biological replicate. Six biological replicates per treatment and per time point were used and the whole experiment was conducted one-time. Total RNA was extracted by TRI Reagent^®^ for DNA, RNA and protein isolation (Sigma-Aldrich) according to the manufacturer’s instructions. RNA concentration and purity were detected spectrophotometrically (8453, Agilent, USA). DNase treatment was performed by RQ1 RNase-free DNase (Promega). RNA was converted to cDNA using 6 mg of DNA-free RNA, 3 µL of 100 nm random nanomers and 4 µL of RevertedAid^TM^ M-MuLV reverse transcriptase (Fermentas, Lithuania).

qPCR was conducted with HS-qPCR SYBR Blue (2×) mix (BioLabMix, Russia) in a CFX96 Touch system (Bio-Rad, USA). qPCR was performed in three technical replicates under the following conditions: 95 °C for 3 min; followed by 40 cycles of 94 °C for 15 s and annealing and elongation at 62/60/57 °C, depending on the primers (Appendix B, Table S2), for 30 s; and generation of melting curves (70–90 °C). The quality of the PCR products was verified by gel electrophoresis. The melting curves for each sample were analyzed after each run to check the specificity of amplification. Gene expression was calculated by the ΔΔCq protocol with Bio-Rad CFX Manager software (Bio-Rad). Two genes of *Galleria mellonella* were used as reference genes: translation elongation factor 1-alpha (EF1a) and 11 subunit of eukaryotic DNA-dependent RNA polymerase II (RBP11). Eight genes of interest (gloverin, gallerimycin, galiomycin, IMPI, Hsp 90, Contig 15362, Contig 17373, and Contig 20582) were investigated. Primer sequences are provided in Appendix B, Table S2. Primer properties were estimated by IDT OligoAnalyzer 3.1 (http://eu.idtdna.com/calc/analyzer).

### Bioassay of culturable bacteria

Three-day-old colonies of *Enterococcus faecalis* (isolate GC1), *Enterobacter* sp. (isolate GB23) and *Serratia marcescens* (isolate GS46) were suspended in sterile 0.1 M phosphate buffer, and the concentrations were diluted to 1.0 × 10^8^ propagules/mL. The suspensions were mixed with wax moth food and placed in Petri dishes (1 mL of suspension per 3 g of food per Petri dish). For the control variants, the food was treated with 1 M phosphate buffer. Fourth-instar larvae were dipped in a water-Tween suspension of *B. bassiana* (4.0 × 10^6^ conidia/mL) for 10 s and placed in Petri dishes (10 larvae per dish) with treated food and moistened disks of filter paper. Control larvae were treated with the water-Tween solution. Petri dishes were maintained at 28 °C and 75% RH. Mortality was monitored for 10 days. Mummified insects were placed in moisture chambers to establish the cause of mortality. At least three replicates of each treatment were used, and the whole experiment was repeated three times.

### Statistics

Data analyses were performed using SigmaStat 3.1 (Systat Software Inc., USA), Statistica 8 (StatSoft Inc., USA), PAST 3^77^ and AtteStat 12.5^78^. As the distribution of the studied parameters deviated from normal (Shapiro–Wilk test, P < 0.05), we used the nonparametric equivalent of a two-way ANOVA, namely, the Scheirer-Ray-Hare test^79^, followed by Dunn’s post hoc test. Differences in the mortality of wax moth larvae after various treatments were analyzed by the *χ*^2^ test. Differences in survival durations were estimated by the log-rank test followed by the Holm-Sidak adjustment. The synergistic and additive effects after combined treatments were differentiated by comparing the expected and observed mortality rates using the χ^2^ test, as suggested by Robertson and Preisler^80^.

## Supplementary information

Supplementary figures and tables

Dataset 1
