# Supplementary material for: Parasitoid envenomation alters the Galleria mellonella midgut microbiota and immunity, thereby promoting fungal infection
Source: Sci Rep. 2019 Mar 8;9:4012. doi: 10.1038/s41598-019-40301-6 (PMC6408550; doi:10.1038/s41598-019-40301-6)
Supplement: Supplementary file 1 — Supplementary figures and tables [file 41598_2019_40301_MOESM1_ESM.pdf]

Appendix B

Parasitoid envenomation alters the *Galleria mellonella* midgut microbiota and immunity, thereby promoting fungal infection

Olga V. Polenogova, Marsel R. Kabilov, Maksim V. Tyurin, Ulyana N. Rotskaya, Anton V. Krivopalov, Vera V. Morozova, Kseniya Mozhaitseva, Nataliya A. Kryukova, Tatyana Alikina, Vadim Yu. Kryukov, Viktor V. Glupov

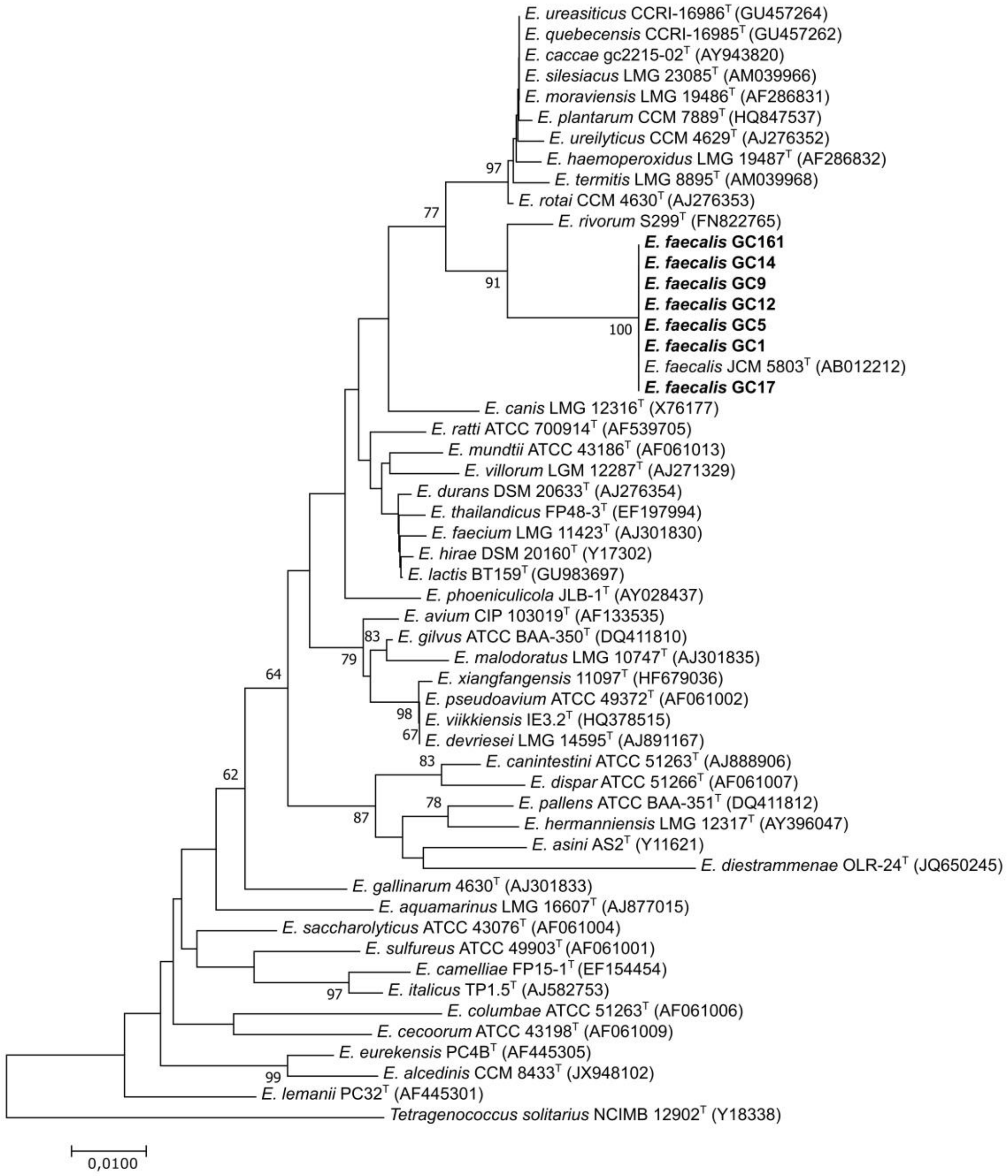

Figure S1. Phylogenetic analysis of the investigated 16S rRNA gene sequences of cultivable *Enterococcus* strains from *Galleria mellonella* midguts, along with the most similar sequences. The GenBank identifiers (gi) for the sequences are shown in parentheses. The neighbor-joining method and the maximum composite likelihood model were used to construct the trees. Bootstrap statistical support (1000 replicates) of greater than 60% is shown at the nodes. The sequence IDs of the investigated bacterial strains are in bold font.

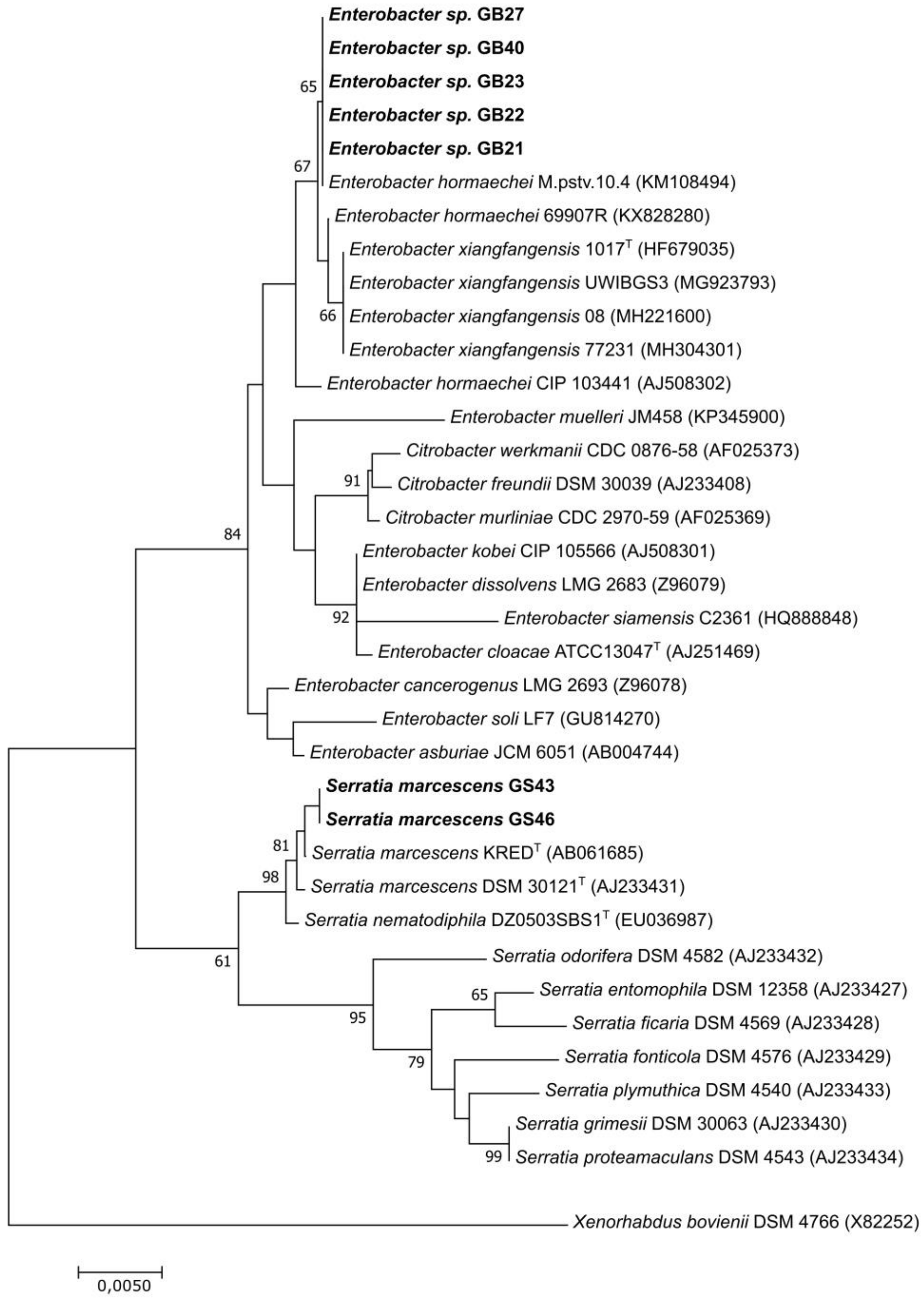

Figure S2. Phylogenetic analysis of the investigated 16S rRNA gene sequences of cultivable *Enterobacter* and *Serratia* strains from *Galleria mellonella* midguts, along with the most similar sequences. The GenBank identifiers (gi) for the sequences are shown in parentheses. The neighbor-joining method and the maximum composite likelihood model were used to construct the trees. Bootstrap statistical support (1000 replicates) of greater than 60% is shown at the nodes. The sequence IDs of the investigated bacterial strains are in bold font.

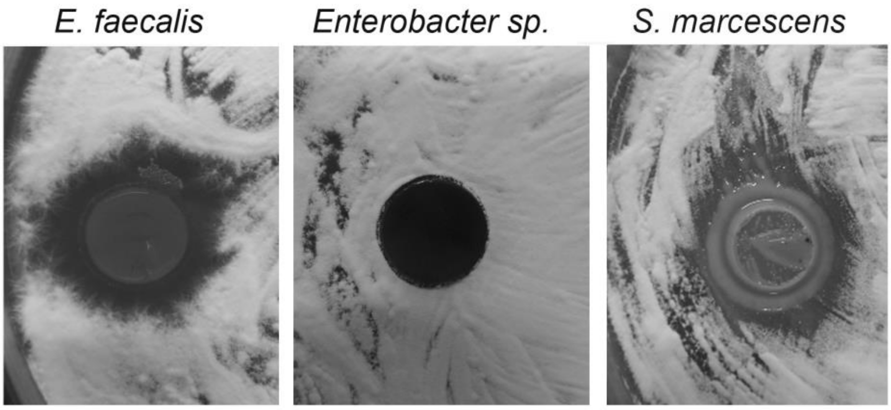

Figure S3. Inhibition of *Beauveria bassiana* mycelial growth by bacteria isolated from wax moth midguts. The diameter of the plugs is 8 mm.

Table S1. Expected and observed mortality of wax moth larvae after treatment with *B. bassiana* (Bb) and cultivable midgut bacteria.

	Days after treatment									
	1	2	3	4	5	6	7	8	9	10
**Bb and Entrococcus**										
Expected mortality, %	0	1,39	7,65	15,3	20,7	35	38,1	42,1	47,4	52,1
Observed mortality, %	0,67	1,33	24,1	35,4	42,7	54,7	66,2	67,8	73,5	77,3
Observed – Expected	0,67	-0,1	16,4	20,1	22	19,8	28,1	25,7	26,1	25,2
χ^2	–	0	**58,6***	**48**	**45,3**	**26,4**	**51,7**	**41,6**	**42,2**	**39,1**
**Bb and Entrobacter**										
Expected mortality, %	0	1,39	7,65	15,3	20,7	35	38,1	42,1	47,4	52,1
Observed mortality, %	0	0	20,7	32,5	39,8	53,3	64	68,8	70,9	75,2
Observed – Expected	0	-1,4	13,1	17,1	19,1	18,3	25,9	26,6	23,5	23
χ^2	–	2,42	**41,7**	**38,9**	**38**	**25,3**	**49,1**	**50,1**	**38,1**	**36,6**
**Bb and Serratia**										
Expected mortality, %	0	0,63	6,09	14,9	20,3	33,8	36,9	42,1	47,3	51,4
Observed mortality, %	0	0	19,9	28,8	43,1	66,8	74,7	77,1	81,2	83
Observed – Expected	0	-0,6	13,8	14	22,9	33,1	37,8	35	33,9	31,7
χ^2	–	1,12	**59,2**	**27,4**	**57,5**	**87**	**109**	**89,6**	**82,2**	**71,5**

* - Values in bold font show significant synergistic effects ($\chi^2 > 3.84$, df = 1, $P < 0.05$) when analyzed as described by Robertson and Preisler [4].

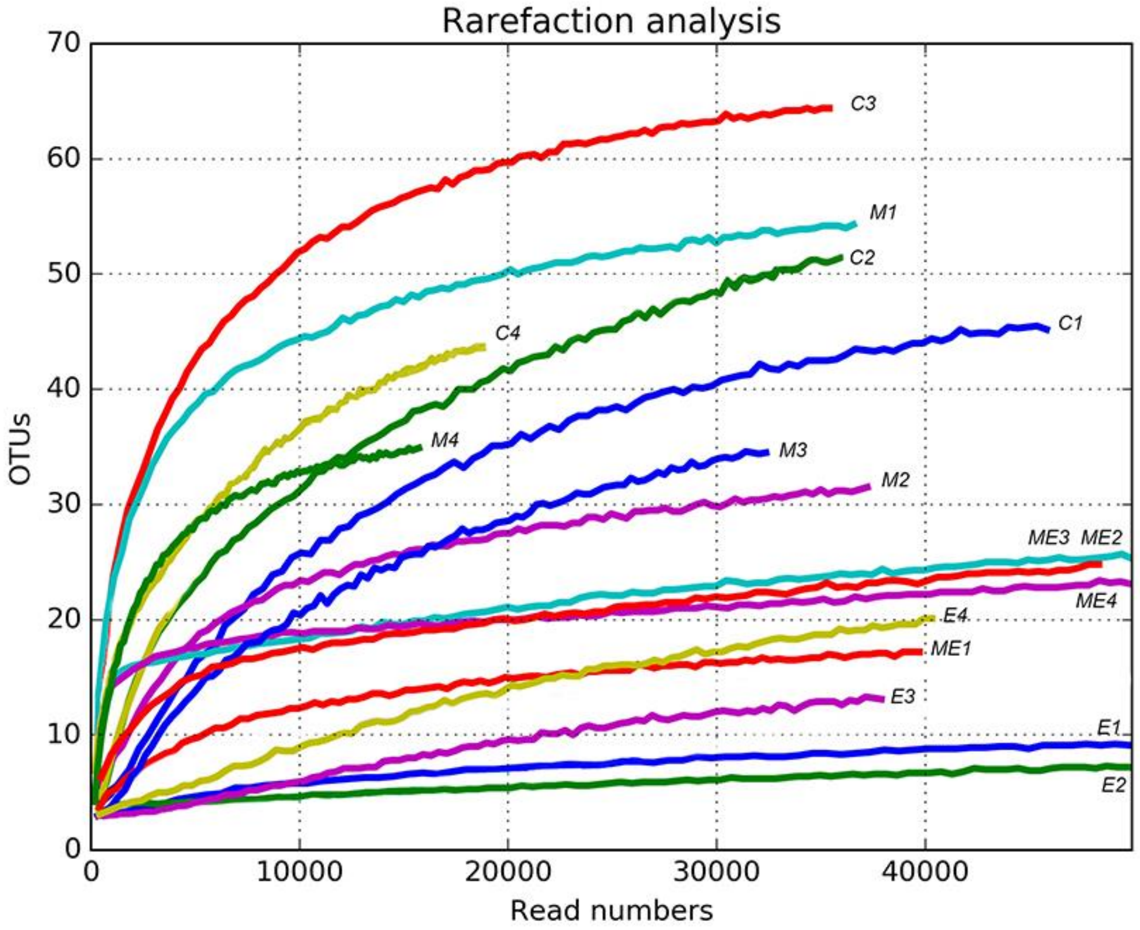

Figure S4. Rarefaction curves of the OTU number for each sample. *C* – control; *M* – mycosis; *E* − envenomation; *EM* − mycosis and envenomation; *1, 2, 3, 4* − replicates.

Table S2. List and description of genes and primers sequences used in the qPCR.

Gene Name	NCBI genebank Accession number	Gene symbol	Primer sequence (5' – 3')	Product size (bp)	PCR efficiency (±SD)	Tm in qPCR, °C	Primers source
Translation elongation factor 1-alfa	AF423811.1	EF1a	For CTG(AACCTCCTTACAGTGAATCC)*<br>Rev GC(ATGTTATCTCCGTGCCAG)*	135(127)	2,05±0,06/<br>2,04±0,02/<br>(2,02±0,04)	62/60/(57)	Melo et al., (2013) [b]
11 subunit of eukaryotic DNA-depended RNA polymerase II	NTHM01000029.1 (wgss)<br>GME-string_Contig_515.0[a]	RBP11	For CGCCAACCTTTGAATCATTCCTT<br>Rev TGGTGTCTGATCATGTTTCCAAGA	136	1,92±0,08/<br>1,96±0,04/<br>1,93±0,06	62/60/57	ISEA[c]
Gallerimycin	AF453824.1	Gal	For GAAGTCTACAGAATCACACGA<br>Rev ATCGAAGACATTGACATCCA	161	1,92±0,4	62	Melo et al., (2013) [b]
Gloverin	AF394588.1	Glo	For AGATGCACGGTCCTACAG<br>Rev GATCGTAGGTGCCTTGTG	93	2,00±0,06	62	Melo et al., (2013) [b]
Galiomycin	AY528421.1	Glm	For GTGCGACGAATTACACCTC<br>Rev TACTCGCACCAACAATTGAC	103	2,02±0,03	62	Melo et al., (2013) [b]
Inducible metalloproteinase inhibitor	AY330624.1	IMPI	For TAGTAAGCAGTAGCATAGTCC<br>Rev GCCATCTTCACAGTAGCA	161	2,04±0,04	57	Melo et al., (2013) [b]
Heat shock protein 90	AF394591.1	Hsp90	For TCAGCTTCACGGACAGCTTCT<br>Rev GACCCCAGAGCTTGCATTGG	152	2,03±0,05	62	ISEA[c]
Caspase-like protein	NTHM01000022.1 (wgss)<br>contig15362_1[a]	CASP	For AGCTACATCCCAGAGGATTCA<br>Rev TCTTCTGTGGGGCAGTCA	104	2,01±0,03	60	ISEA[c]
Glutathione peroxidase	NTHM01000062.1 (wgss)<br>contig17373_1[a]	GSH PX	For CCACACTGTGAGGCAACATT<br>Rev GTTTGCTTAGCACGGTCACA	188	1,99±0,07	62	Melo et al., (2013) [b]
Macrophage migration inhibitory factor	NTHM01000005.1 (wgss)<br>contig20582_1[a]	MIF	For AACAGTACTGCGTGGTGA<br>Rev TTCCACGCCAAGGGATCCAA	108	2,03±0,04	60	ISEA[c]

* round brackets denote modifications for Tm = 57 °C

[a] Vogel and coauthors[1]

[b] Melo and coauthors[2]

[c] Primers were designed in the Laboratory of Insect Pathology ISEA SB RAS with an online resource, https://www.ncbi.nlm.nih.gov/tools/primer-blast/, and their properties were estimated by IDT OligoAnalyzer 3.1 (http://eu.idtdna.com/calc/analyzer).

References

1. Vogel, H., Altincicek, B., Glöckner, G. & Velcinskas, A. A comprehensive transcriptome and immune-gene related repertoire of the lepidopteran model host *Galleria mellonella*. *BMC Genomics* **12**, 308, (2011).
2. Melo, N.R., Abdrahman, A., Greig, C., Mukherjee, K., Thornton, C., Ratcliffe, N.A., Vilcinskas, A. & Butt, M.T. Myriocin significantly increases the mortality of a non-mammalian model host during *Candida* pathogenesis. *PlosOne*, 2013. DOI: 10.1371/journal.pone.0078905.
3. Kumar S, Stecher G, Tamura K (2016) MEGA7: Molecular Evolutionary Genetics Analysis 7.0 for bigger datasets Molecular Biology and Evolution 33:1870-1874.
4. Robertson JL and Preisler HK. Pesticide Bioassays with Arthropods. CRC. Boca Raton. FL (1982).
